# Supplementary material for: Bim escapes displacement by BH3-mimetic anti-cancer drugs by double-bolt locking both Bcl-XL and Bcl-2
Source: eLife. 2019 Mar 12;8:e37689. doi: 10.7554/eLife.37689 (PMC6414199; doi:10.7554/eLife.37689)
Supplement: Transparent reporting form [file elife-37689-transrepform.pdf]

eLife's transparent reporting form

We encourage authors to provide detailed information *within their submission* to facilitate the interpretation and replication of experiments. Authors can upload supporting documentation to indicate the use of appropriate reporting guidelines for health-related research (see EQUATOR Network), life science research (see the BioSharing Information Resource), or the ARRIVE guidelines for reporting work involving animal research. Where applicable, authors should refer to any relevant reporting standards documents in this form.

If you have any questions, please consult our Journal Policies and/or contact us: editorial@elifesciences.org.

Sample-size estimation

- You should state whether an appropriate sample size was computed when the study was being designed
- You should state the statistical method of sample size computation and any required assumptions
- If no explicit power analysis was used, you should describe how you decided what sample (replicate) size (number) to use

Please outline where this information can be found within the submission (e.g., sections or figure legends), or explain why this information doesn't apply to your submission:

Not applicable. The study uses automated collection of large numbers of datapoints and a minimum of three independent replicates for each experiment.

Replicates

- You should report how often each experiment was performed
- You should include a definition of biological versus technical replication
- The data obtained should be provided and sufficient information should be provided to indicate the number of independent biological and/or technical replicates
- If you encountered any outliers, you should describe how these were handled
- Criteria for exclusion/inclusion of data should be clearly stated
- High-throughput sequence data should be uploaded before submission, with a private link for reviewers provided (these are available from both GEO and ArrayExpress)

Please outline where this information can be found within the submission (e.g., sections or figure legends), or explain why this information doesn't apply to your submission:

Figure 1 illustrates how TCSPC data were collected and analyzed. For the sample data analysis it is indicated in Figure 1b that the binding curves shown were generated from TCSPC data for more than 8,500 regions of interest (ROIs) in FLIM micrographs from at least three separate experiments. The number of ROIs in each bin is indicated in the figure using a greyscale. No outliers were removed but, as stated, a bin was not created for less than 20 data points. Individual data points are shown for data regions with less than 20 data points but were not used in the calculations.

Inclusion/exclusion criteria: In addition on pages 50 (lines 1-15) we describe additional criteria for selection of ROIs and calculation of binding curves.

In the caption for Figure 1d we state "***In all figures FLIM-FRET binding curves as shown in Figure 1d-f and figure supplements were fit from data pooled from 3 independent experiments. Individual points in FLIM-FRET binding curves indicate the average FLIM-FRET efficiencies in corresponding bins (the number of data points ranges from 20 to 3,000 in each bin), the error bars indicate standard error of the mean, and the dotted shadowed area for each curve represents the 95% confidence interval for the fit of the binding curve with a Hill slope to the data as in (b).Truncation of some of the curves is due to a lack of sufficient data at high expression levels of the acceptor proteins. This can be due to induction of apoptosis by the expressed protein or to limited expression of the exogenous protein in the transient transfections.***" This is restated in less detail in other figure captions. For fitting the data with a standard binding curve with a Hill slope, data was selected as described on page 50. We also indicate on Page 49 lines 13-20 that in some experiments (Figure 4 – Figure supplement 4c) the data at ratios of Venus to mCerulean3 greater than 0.5 are not well described by a standard binding curve. For this reason quantitative comparisons were restricted to those regions of the curves that are fit using a standard binding curve with a Hill slope. For vtBid and vBad it was not possible to measure binding to cBcl-XL at ratios of Venus to mCer3 intensities greater than ~ 0.7 in the presence of ABT-263 because the released vBH3-proteins killed the cells. This is also the reason that the means for each bin and the resulting binding curves are provided for all of the figures as supplements. For FLIM data an independent experiment is a separate transfection carried out on a different day.

Cell Free experiments: The captions for Figures 2e, 5c-e indicate that for cell free data using purified proteins and liposomes or mitochondria individual data points for three replicates are shown (although they are not all visible because they overlap). This also applies to data for F/Fo for NBD fluorescence in Figure 9 as described in the caption. For Figure 7d contain too many datapoints to show all and therefore each point is represented as a mean +/- std dev as stated in the figure caption. Experimental replicates may use the same batches of purified proteins but the experiments are carried out independently. Cell free data indicate that the number of experimental replicates was three.

For the cell based experiments for the function of the fusion proteins reported in Figures 3 and 5 we report in the methods on page 46 that the number of cells for which measurements of mitochondrial transmembrane potential, nuclear and cell area were acquired was at least 1000 for each replicate for each cell death curve. In the caption for Figure 3 we report that each data point represents three averages from three independent experiments with each replicate including more than 30 cells for each intensity bin. Each replicate is a separate transfection carried out on a different day. All cell data was included, outliers were not encountered. We also state that for each experiment > 100 positive and >100 negative control cells were used to train the classifier.

eLife Sciences Publications, Ltd is a limited liability non-profit non-stock corporation incorporated in the State of Delaware, USA, with company number 5030732, and is registered in the UK with company number FC030576 and branch number BR015634 at the address 1st Floor, 24 Hills Road, Cambridge CB2 1JP | August 2014

For Colocalization analyses the analysis pipeline is documented in detail on page 51. This pipeline description includes details for data exclusion: “Objects suitable for analysis were identified by mean Venus intensity to identify cells expressing appropriate levels of the Venus-tagged proteins of interest. A median Venus intensity (low threshold) was used to discard any cells that passed the mean Venus filter due to improper segmentation (ie. overlap between an untransfected cell and a bright transfected cell). Objects were further selected by mean MitoTracker-Red intensity (high and low threshold), to ensure appropriate staining. A size filter was used to remove small dead cells or debris and any abnormally large (possibly improperly segmented) cells”.

Statistical reporting

- Statistical analysis methods should be described and justified
- Raw data should be presented in figures whenever informative to do so (typically when N per group is less than 10)
- For each experiment, you should identify the statistical tests used, exact values of N, definitions of center, methods of multiple test correction, and dispersion and precision measures (e.g., mean, median, SD, SEM, confidence intervals; and, for the major substantive results, a measure of effect size (e.g., Pearson's r, Cohen's d)
- Report exact p-values wherever possible alongside the summary statistics and 95% confidence intervals. These should be reported for all key questions and not only when the p-value is less than 0.05.

Please outline where this information can be found within the submission (e.g., sections or figure legends), or explain why this information doesn't apply to your submission:
Figure 1 - For visualization the distribution of data within bins is shown as box and whisker plots as defined in the figure caption.
For all figures the FLIM means were fit to a binding curve with a Hill slope using GraphPad Prism. The caption for figure 1 includes the statement: "Individual points in FLIM-FRET binding curves indicate the average FLIM-FRET efficiencies in corresponding bins (the number of data points ranges from 20 to 3,000 in each bin), the error bars indicate standard error of the mean, and the dotted shadowed area for each curve represents the 95% confidence interval for the fit of the binding curve with a Hill slope to the data". This statement applies to all FLIM data in the paper. The number of TCSPC photon counts per ROI was in excess of 1000 as reported on page 47 lines 8-10.

In figure 2 we provide an illustration for how R values were calculated with explicit instructions for the calculation in the caption. At the end of the caption fir Figure 1 we indicate that R values are presented with error bars derived from the 95% confidence interval from the model fit. This is reiterated in the captions of other Figures.The rationale and limitations imposed by the use of R values is described on page 49-40.

All of the binding curves used to calculate the R values are presented in the supplements for the corresponding figures.

The numerical values for all of the R values in all of the figures are presented in Table 1 along with the 95% confidence intervals for each (page 13 of the text). We refrain from claims of statistical significance and instead provide the R values, confidence intervals and binding curves for the reader.

The statistical analysis of cell free data using purified proteins is reported in the caption for Figure 2e. Independent experiments were performed in triplicate with all data points plotted. The individual data points are all shown but are not always visible because they overlap.

In Figure 7d-e individual data points are not shown because the means were used to fit the binding curves shown and the overlap between three of the curves is such that adding additional data points would not improve the utility of

the figure. The figure caption states that data are mean ±SD (n=3 independent experiments).

In Figure 9 values for Ksv are shown with 95% confidence intervals for linear fitting of the data as described in the caption. The data used to fit the models shown in the figure supplement comes from three independent experiments and is shown as means and standard deviations for visualization. The raw data was used to calculate values for Ksv as indicated in the caption on page 38.

Cell death data are stated as being comprised of more than 1000 cells per dose response curve for each replicate. Each data point represents the average values for the three replicates and therefore the error is SEM for the three means used to generate each point. On page 17 the statistical analysis for Figure 3 states in the caption: “Data (% Dead) for three independent replicates (circles) and the mean of the replicates (line) are plotted. A one-way ANOVA test was performed with a Dunnett's Multiple Comparison post-test (Graphpad Prism), to compare all treated wells with untreated controls for each cell line.” The assumption made for this test is that all source data has common standard deviation. That this is approximately correct can be seen in the data presented as the independent replicates are plotted.

Values for Pearson’s R are provided as means of values calculated on a per cell basis from more than 150 cells with a minimum of 20 cells in each replicate. Means were calculated from the averages obtained for each replicate therefore error bars are +/- SEM as stated in the caption to Figure 8. As the utility of this figure is to compare trends between binding measurements and localization, presenting individual data points would not be helpful.

To test correlation between localization and Resistance to ABT-263 the data was analyzed in aggregate using Spearmans rank-order correlation to include relationships that might not be linear. Furthermore, Spearman's rank order does not assume a relationship at the start of the analysis.The results of this test indicating there was no correlation between localization for Bcl-XL and a weak correlation for Bcl-2 is reported on page 33.

(For large datasets, or papers with a very large number of statistical tests, you may upload a single table file with tests, Ns, etc., with reference to sections in the manuscript.)

Group allocation

- Indicate how samples were allocated into experimental groups (in the case of clinical studies, please specify allocation to treatment method); if randomization was used, please also state if restricted randomization was applied

- Indicate if masking was used during group allocation, data collection and/or data analysis

Please outline where this information can be found within the submission (e.g., sections or figure legends), or explain why this information doesn't apply to your submission:

None of the data is grouped.

Additional data files ("source data")

- We encourage you to upload relevant additional data files, such as numerical data that are represented as a graph in a figure, or as a summary table
- Where provided, these should be in the most useful format, and they can be uploaded as "Source data" files linked to a main figure or table
- Include model definition files including the full list of parameters used
- Include code used for data analysis (e.g., R, MatLab)
- Avoid stating that data files are "available upon request"

Please indicate the figures or tables for which source data files have been provided:

We have provided the binding curves from which the R values were derived. The data for the binding curves has been provided as source data in tabular form. At present the repository for microscopy data (the Open Microscopy Environment - OME) does not accept FLIM micrographs. This paper reports data derived from more than 9000 micrographs of 256X256 pixels, each pixel of which includes intensity data and photon arrival times for hundreds to thousands of photons in a proprietary format. It would not be useful to upload this data until OME is prepared to handle it. I am in conversation with Jason Swedlow to accomplish this whereupon we will upload our FLIM data. The cell death data summarizes more than 3 million data points. Individual dose response curves represent at least 1000 data points. For colocalization data mean values represent at least 20 data points. Means of for the three replicates represent at least 150 data points.
